# Zebrafish Is a Powerful Tool for Precision Medicine Approaches to Neurological Disorders

**DOI:** 10.3389/fnmol.2022.944693

**Published:** 2022-07-06

**Authors:** Katarzyna Ochenkowska, Aveeva Herold, Éric Samarut

**Affiliations:** ^1^Centre de Recherche du Centre Hospitalier de l’Université de Montréal, Montreal, QC, Canada; ^2^Department of Neuroscience, Université de Montréal, Montreal, QC, Canada; ^3^Modelis Inc., Montreal, QC, Canada

**Keywords:** precision medicine, neurological disorders, zebrafish, functional genomics, drug discovery

## Abstract

Personalized medicine is currently one of the most promising tools which give hope to patients with no suitable or no available treatment. Patient-specific approaches are particularly needed for common diseases with a broad phenotypic spectrum as well as for rare and yet-undiagnosed disorders. In both cases, there is a need to understand the underlying mechanisms and how to counteract them. Even though, during recent years, we have been observing the blossom of novel therapeutic techniques, there is still a gap to fill between bench and bedside in a patient-specific fashion. In particular, the complexity of genotype-to-phenotype correlations in the context of neurological disorders has dampened the development of successful disease-modifying therapeutics. Animal modeling of human diseases is instrumental in the development of therapies. Currently, zebrafish has emerged as a powerful and convenient model organism for modeling and investigating various neurological disorders. This model has been broadly described as a valuable tool for understanding developmental processes and disease mechanisms, behavioral studies, toxicity, and drug screening. The translatability of findings obtained from zebrafish studies and the broad prospect of human disease modeling paves the way for developing tailored therapeutic strategies. In this review, we will discuss the predictive power of zebrafish in the discovery of novel, precise therapeutic approaches in neurosciences. We will shed light on the advantages and abilities of this *in vivo* model to develop tailored medicinal strategies. We will also investigate the newest accomplishments and current challenges in the field and future perspectives.

## Introduction

Precision medicine is one of the so-called “hot topics” in applied sciences, modern biomedicine, and biomedical studies. However, the term “precision medicine” is an umbrella for a vastness of definitions that could make its understanding complex, imprecise and somehow confusing. As a generally acknowledged definition, precision medicine, also known as personalized medicine, is a form of medicine that considers individual variability in genes, environment, and lifestyle to prevent, diagnose or treat a disease. As per the Centers for Disease Control and Prevention CDC, “*precision medicine helps doctors find unique disease risks and treatments that will work best for patients*.” Some may think that personalized medicine is an invention of the 21st century. However, in the light of these general definitions, this facet of medicine and human sciences is not new. Indeed, since the dawn of time, physicians have always tailored their medical recommendations to individual factors such as age, gender, and other patients’ lifestyle specificities. First reports of adapting medicine to an individual’s health status are found in the history of ancient Egypt, where the medicine was divided into categories by different body parts (Visvikis-Siest et al., 2020). Then, with the rise of modern medicine in the 20th century, precision medicine relied on more accurate molecular clues such as rhesus factors defining blood groups (Klein et al., 2015). Thus, it quickly became practical to group patients based on their blood type to improve successful blood transfusions. The *personalized* aspect of medicine became more tangible at the beginning of the 21st century following human genome sequencing. Indeed, it is now possible to correlate the genetic fingerprint of individuals to their general health and treatment responsiveness (Brittain et al., 2017; Carrasco-Ramiro et al., 2017). This is the basis of the modern definition of precision medicine that aims to improve diagnosis and prognosis and tailor the medication to an individual based on genetic variations.

Because a simple definition of precision medicine cannot quickly be drawn, it seemed essential to us to start this review by determining three main pillars of precision medicine we will be discussing here: (1) Predicting disease susceptibility, progression, and improving diagnosis, (2) Accelerating drug discovery; (3) Predicting treatment-responsiveness and eliminating trial-and-error inefficiencies of current medical plans. Importantly, these main missions of precision medicine can be applied to a broad list of biomedical disciplines, including oncology (Krzyszczyk et al., 2018; Gambardella et al., 2020; Malone et al., 2020), immunology (Boyd et al., 2017; Ballow and Leiding, 2021; Distler et al., 2021), and neurological disorders (Freudenberg-Hua et al., 2018; Schneider and Alcalay, 2020; Striano and Minassian, 2020).

Although we have just stated that precision medicine is not a novel aspect of science, research investigating the correlation of genetic factors to medical outcomes is still in its infancy. Such translational research relies on developing predictable, reproducible, and reliable animal models in which genotype to phenotype correlations can be accurately assessed. Moreover, these models must be amenable to pharmacological studies and drug screening approaches. Zebrafish is a well-known model for investigating biological issues, particularly central nervous system (CNS) development (Blader and Strahle, 2000; Schmidt et al., 2013). Embryos are convenient for genetic manipulations, including CRISPR-CAS9 genome engineering (Hwang et al., 2013) and the development of CNS structures can be followed *in vivo* at a single-cell resolution thanks to an extensive repertoire of available transgenic lines (Park et al., 2000; Zerucha et al., 2000; Shin et al., 2003; Kimura et al., 2006). Zebrafish have an integrated nervous system, and the brain of a zebrafish larvae already contains homologous brain structures to those found in mammals (Vaz et al., 2019; Corradi and Filosa, 2021) as well as equivalent cellular and synaptic networks and functions (Kaslin and Panula, 2001; McLean and Fetcho, 2004; Filippi et al., 2010; Panula et al., 2010). Furthermore, the zebrafish embryo or larva shows a complex behavioral repertoire as early as a few days post-fertilization (Tegelenbosch et al., 2012). Finally, being favorable to preclinical drug discovery, zebrafish is a model of choice for pharmacological studies.

In this review, we will limit ourselves to the development of precision medicine approaches in the context of neurosciences. Particularly, we will discuss the predictive power of zebrafish in the discovery of novel, precise therapeutic approaches in this framework. We will shed light on the advantages and abilities of this *in vivo* model to develop tailored medicinal methods. Mainly, we will discuss how it can participate in the mission of the three pillars of precision medicine we described above. We will also investigate the newest accomplishments and current challenges in the field and future perspectives it could offer.

## Using Zebrafish to Predict Neurological Disease Susceptibility and Progression and Improve Diagnosis

### Unraveling Pathogenic Molecular Mechanisms by Functional Genomics Using Zebrafish

The study of the mechanisms that predispose, cause, or participate in the development of neurological pathologies can be enlightened through functional genomics approaches using adequate experimental models. As the name suggests, *functional genomics* aims at deciphering the function of genes and their role in a genuine biological system (healthy and unhealthy). A few decades ago, complete genome sequencing provided a framework for comprehensively investigating biological processes (Hocquette, 2005; Bunnik and Le Roch, 2013). Functional genomics integrates molecular biology and cell biology studies to explore the whole structure, process, and regulation of genes of interest to examine the function of a myriad of genes with unknown parts. Even if the causative gene is identified for many genetic diseases, the molecular pathogenic substratum often remains unknown. This issue is more critical in the context of rare genetic neurological disorders since they are understudied and not well-understood. It is essential to unravel the molecular mechanisms underlying the disease phenotype as they could identify actionable targets for further therapeutic development.

The zebrafish model is an excellent experimental approach for such functional investigations as a valuable scientific tool. Indeed, the latest improvements in bioengineering techniques allow researchers to study the functions of genes and the impact of their mutations directly *in vivo* in zebrafish larvae. As vertebrates, zebrafish, and human genomes show a high homology, about 80% of genes associated with diseases in patients are conserved in zebrafish (Kalueff et al., 2014). Notably, many CNS-related disorders have been successfully modeled in the past and some recent reviews have compiled an exhaustive list of zebrafish models of Amyotrophic Lateral Sclerosis (ALS) (Braems et al., 2021), Hereditary Spastic Paraplegia (HSP) (Naef et al., 2019; Quelle-Regaldie et al., 2021a,b), Epilepsy (Rosch et al., 2019; Gawel et al., 2020), Autism Spectrum Disorder (ASD) (Meshalkina et al., 2018; de Abreu et al., 2020), Alzheimer’s Disease (AD) (Saleem and Kannan, 2018), Parkinson’s Disease (PD) (Unal and Emekli-Alturfan, 2019; Najib et al., 2020), Huntington’s and Prion-related diseases (Wang et al., 2021), Serotonin syndrome (SS) (Stewart et al., 2013), and Glioblastoma (Reimunde et al., 2021). In this context, our group pioneered the generation of several models of CNS genetic disorders, caused by mutations in *gabra1* (Samarut et al., 2018), *gabrg2* (Liao et al., 2019), *depdc5* (Swaminathan et al., 2018), *glra1* (Samarut et al., 2019), or *gldc* (Riche et al., 2018), and these mutants display clinically-relevant phenotypes such as seizures, ataxic motor phenotypes or hypotonia. More importantly, studies at the cell network and molecular levels using these zebrafish genetic models have made it possible to highlight novel aspects of the underlying pathogenicity. For instance, mimicking DEPDC5 (DEP containing 5 domain) loss-of-function in zebrafish recapitulates critical hallmarks of brain disorders caused by mutations in this gene, such as epileptiform discharges and exacerbated mTOR signaling (de Calbiac et al., 2018; Swaminathan et al., 2018). However, a closer examination of neural cell networks *in vivo* revealed a drastic reduction in the number and complexity of inhibitory synapses in the brain of *depdc5-/-* zebrafish larvae compared to their siblings. More excitingly, pharmacological studies showed that this phenotype is not caused by a defect in the classical function of the *DEPDC5* gene (e.g., mTOR inhibitor) but is rather mTOR-independent. This study, therefore, opens a yet undiscovered aspect of DEPDC5 biology and related brain disorders. In the same line of thought, modeling loss-of-function in a gene involved in glycine breakdown (glycine decarboxylase, *GLDC*) in zebrafish leads to an increase in the accumulation of glycine in tissues and to premature death, as is the case in corresponding mouse models (Narisawa et al., 2012; Pai et al., 2015; Riche et al., 2018). However, thanks to the accessibility of zebrafish embryos to quantitative molecular techniques, such as whole transcriptome sequencing, it was shown that the metabolic perturbances caused by GLDC loss-of-function in zebrafish go beyond glycine only. Indeed, molecular profiling of key amino-acid metabolites by liquid-chromatography mass-spectrometry (LCMS) in *gldc-/-* zebrafish larvae identified significant changes in the level of several branched-chain amino acids (Riche et al., 2018). Interestingly, other disorders caused by an accumulation of these branched-chain amino acids have been described, and this calls for new potential cross-comparisons between neurometabolic diseases. Interestingly, such exploratory molecular profiling assays *in vivo* in zebrafish models of genetic disorders can also help identify potential novel disease biomarkers that can further be validated in the patient populations.

Finally, it is worth mentioning that zebrafish can also be used to unravel fundamental aspects of pathologies without necessarily necessitating the use of genetic models. Indeed, thanks to its optical transparency and an extensive repertoire of available transgenic lines (Park et al., 2000; Zerucha et al., 2000; Shin et al., 2003; Kimura et al., 2006), zebrafish are convenient to follow, *in live*, brain activity under specific pharmacological exposures. For example, Diaz-Verdugo et al. studied the respective roles of non-neuronal glial cells vs. neurons in the brain state transition leading to epilepsy brain seizures (Diaz Verdugo et al., 2019; Rosch et al., 2019). To do this, they elegantly utilize zebrafish larvae to record the activity of both neurons and glial cells following exposure to a proconvulsant GABA-antagonist (pentylenetetrazol or PTZ). Surprisingly, their work revealed a robust and widespread activation of a glia network *before* the generalization of neuronal activity, the latter being characteristic of generalized epileptic seizures. They showed that whereas glial activation before brain seizure seems to reduce synchronous neuronal activity, a collapse of the glial homeostatic regulation may precipitate a generalized release of glutamate in the extracellular space leading to a generalized seizure. This provides a new perspective on seizure spreading in the brain and opens a new door to therapy development targeting non-neuronal cells.

Thanks to its pharmacological and genetic accessibility, the use of see-through zebrafish larvae is a powerful catalyzer for discovering novel pathogenic mechanisms associated with neurological disorders. Such findings are instrumental for accelerating the precision medicine mission that aims to predict disease susceptibility and prevent its progression. Although these studies strongly advocate for the translational potential of CNS investigations in zebrafish, one must keep in mind that there are some important differences in the development of certain brain areas between zebrafish and mammals. Many structures in the zebrafish brain can be considered neuroanatomical similar to mammalian ones, however, they somehow display morphological differences in their development (Friedrich et al., 2010; Cheng et al., 2014). Moreover, as for many non-primate animal models, zebrafish is lacking a canonical hippocampus or cortex. As a result, these developmental and/or morphological discrepancies must be taken into account when modeling brain diseases in zebrafish.

### Validating Genetic Variants Associated With Neurological Disorders Using Zebrafish

One of the main current challenges in precision medicine is improving and accelerating disease diagnosis. Next-generation sequencing radically changed biomedicine by identifying loci, genes and associated mutations involved in specific diseases (Phan et al., 2006). These new genetic data are instrumental for discovering the etiology of human diseases, and the number of new disease-causing genetic variants exploded (Koboldt et al., 2013). However, the clinical relevance of genetic information can be limited by the lack of a precise functional characterization. Thus, there is a need to validate the role played by a specific mutation in a simple biological system to infer a pathogenic role. This is particularly relevant in the case when Variants of Unknown Significance (VUS) are identified in patients. These genetic variations for which we do not know the molecular and physiological consequences represent a significant dilemma in genetic diagnosis and genetic counseling (Alosi et al., 2017). Indeed, according to the American College of Medical Genetics and Genomics (ACMG) guidelines, VUS should not be used in clinical decision-making (Richards et al., 2015). Unfortunately, VUS can be predominantly found in sequencing-based clinical genetic tests. For example, in a cross-sectional study of 164 epileptic patients followed by an epileptologist at a Canadian tertiary care centre’s epilepsy clinic, VUS accounted for more than half of the genetic test results (Li et al., 2022). Based on these unpublished data, it is worth noting that if only 10% of the VUS identified could be functionally validated (a rather conservative estimate), this would potentially increase by 70% the overall positivity yield of the genetic testing in this clinical study. Thus, the clinical advantages of genetic testing can be multiplied *via* the simultaneous development of approaches that aim at functionally validating VUS.

Importantly, VUS identified from a targeted genetic panel (rather than from exploratory Whole-Exome-Sequencing) is often a better fit with the patient’s clinical presentation. In those cases, further *in vitro* and *in vivo* functional testing could help confirm, exclude, or guide clinicians toward a diagnosis. Functional characterization is usually performed in research laboratories, and different levels of functional assessment can be achieved from *in vitro* (i.e., recombinant enzyme activity), *in cellulo* (i.e., target gene expression, cellular phenotype) to *in vivo* (i.e., tissue homeostasis and function, behavior). The latter encompassing a higher degree of complexity necessitates fast and complementary *in vivo* approaches that can remain expensive and time-consuming. The development of a platform for the functional characterization of VUS has been shown very successfully in the field of oncology, particularly breast cancers associated with mutations in *BRCA* genes, leading to a significant improvement in the clinical management of cancer patients (Guidugli et al., 2014; Woods et al., 2016; Federici and Soddu, 2020). Another example is the different pathogenic variants in the gene *PALB2* associated with varying levels of risk for breast, ovarian and pancreatic cancers (Boonen et al., 2020). Using a combination of complementary *in vitro* assays, researchers can assess the impact of specific *PALB2* VUS, at the level of individual patients, on the function of the protein in DNA repair, cell cycle regulation and the control of cellular levels of reactive oxygen species (Boonen et al., 2020). This functional characterization of VUS can be valuable for predicting cancer risk and anticipating treatment-responsiveness to cancer therapy for each patient.

**FIGURE 1:**
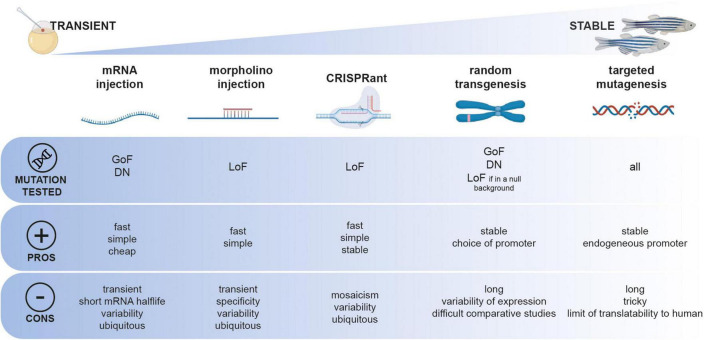
Functional toolbox to study the function of genetic mutation in zebrafish. Different approaches, from transient to stable can be employed and can be used depending on the type of mutation to be tested (GoF, gain-of-function; DN, dominant-negative; LoF, loss-of-function).

Remarkably, simple animal models (i.e., worms, flies, fish) can be sophisticatedly employed to bridge the gap between genetic diagnosis and functional studies. Being compatible with the latest mutagenesis techniques and convenient for deciphering basic pathological mechanisms caused by gene mutations, they can open new avenues for VUS functional characterization. Depending on the class of the genetic mutation of interest to be studied, different functional characterization approaches can be followed using zebrafish (Figure 1). Simplistically, primary pathogenic mechanisms can be divided into a toxic gain of function (GoF) or deleterious loss of function (LoF). Predictably, diseases with a LoF mechanism are inherited in an autosomal recessive manner or an X-linked recessive manner. On the contrary, conditions due to a toxic GoF mechanism are usually inherited autosomal dominant. However, non-exclusive pathogenic LoF and GoF mechanisms can coexist, thus complexifying the study of their pathogenicity in a standardized fashion.

Due to external fertilization, zebrafish embryos can be microinjected at the one-cell stage with *in vitro* transcribed mRNAs for overexpressing a construct of interest. This technique has been exploited for the expression of patient-specific mutations in genes associated with neurological disorders such as Amyotrophic Lateral Sclerosis (Armstrong and Drapeau, 2013) or small-fiber neuropathy (Eijkenboom et al., 2019) as well as for non-neurological conditions such as sinus node dysfunction and atrial fibrillation (Hoffmann et al., 2019). In these studies, clinically relevant phenotypes are assessed in few-day-old larvae, such as swimming behavior, touch-evoked motor response, sensory neurite development or electrophysiologic hallmarks. This transient expression method does not require complex genetic manipulation and can be carried out on large scales and in a short period. However, the experimental variability associated with the manual microinjection of hundreds of embryos and the short half-life of mRNA in the embryo can complicate the interpretation of negative results. To circumvent this problem, other studies took advantage of transposase-mediated stable genomic integration techniques such as Tol2 (Urasaki et al., 2008; Suster et al., 2009) or Sce-I (Hoshijima et al., 2016) random mutagenesis (Figure 1). Researchers generated stable transgenic lines expressing the wild-type or mutant version of a gene of interest, such as the ALS-causative G348C mutation in TDP-43 (Lissouba et al., 2018) or the C1315Y mutation in *COL2A1* associated with lethal fetal skeletal dysplasia (Zhang et al., 2021). Moreover, this technique allows using specific promoter sequences to regulate the expression of the transgene in a tissue- or time-specific fashion. Although this approach has the advantage of not being limited to observing effects solely in the early embryo, it presents one main limitation. Indeed, phenotypes observed upon the stable integration of mutant versions of genes must be carefully compared to their wild-type form. Because of the random nature of these transgenesis techniques, comparing wild-type and mutant allelic versions of a gene of interest that have not been integrated at the same genomic locus and potentially in different copy numbers can be seen as inaccurate. Moreover, the random integration of a transgene of interest remains simulated and cannot accurately be compared to a genuine endogenous expression.

Proving the pathogenicity of loss-of-function mutations can be less challenging, especially in the case of deleterious mutations such as sudden stops or large genomic deletions. In these cases, transient knockdown by morpholino microinjection has been used for years to quickly assess the phenotypic consequence of a specific loss-of-function for *CHD2* in epileptic encephalopathy (Galizia et al., 2015), *CAPN1* in the context of Hereditary Spastic Paraplegia (Gan-Or et al., 2016), ABCC6 in Pseudoxanthoma elasticum (Van Gils et al., 2018), *SBDS* in Shwachman–Bodian–Diamond syndrome (Venkatasubramani and Mayer, 2008), or *VARS* in epilepsy (Siekierska et al., 2019). However, the morpholino-knockdown approach is transient and limited to the first few days of development, and the specificity of morpholinos has also been the subject of debate recently (Stainier et al., 2017). Particularly, strict guidelines have been drawn for the interpretation of morpholino-based assays in zebrafish (Stainier et al., 2017). To overcome these drawbacks, it is now well-admitted in the zebrafish community that a targeted-mutagenesis assay must be preferred, such as Zinc Finger Nucleases (Doyon et al., 2008), TALEN (Hwang et al., 2014), or the popular CRISPR/CAS9 (Hwang et al., 2013; Ran et al., 2013). Targeted mutagenesis tools can also be used for rapid screening in the F0 injected larvae referred as “CRISPRant” using a combination of multi-loci guide RNAs (Kroll et al., 2021). Upon confirmation of a particular phenotype, these injected F0 CRISPRant can be raised and further screened as founders for establishing a stable mutant line. These genetic models can also be used as null *in vivo* genetic backgrounds in which specific genetic variants of the gene of interest can be transiently or stably expressed (mRNA microinjection vs. transposase-mediated random transgenesis). If the wild-type variant can rescue a particular phenotype in a quantitative assay, then it is possible to test the rescuing potential of novel VUS. In that case, the pathogenicity of a specific variant is attributed to the lack of phenotypic rescue. A recent study by Li et al. (2017) from the Harvard Medical School performed a large-scale functional screening of rare genetic variants in the Interferon Regulatory Factor 6 (*IRF6*) gene, potentially associated with orofacial cleft syndromes. They took advantage of a very early-onset embryonic phenotype caused by *irf6* knockout, that is, the improper development of the embryonic epithelium during epiboly, a process occurring only a few hours post-fertilization in zebrafish. The authors used this early rescue assay to test the protein functions of more than 30 human *IRF6* missense variants. Remarkably, they assessed the ability of each genetic variant to rescue the early epiboly defects described in *irf6-/-* embryos through mRNA microinjections at the one-cell stage. Interestingly, when comparing their functional testing results with computational pathogenicity prediction systems (PolyPhen-2 and SIFT), they confirmed the pathogenicity of variants classified as “pathogenic” but found discrepancies in interpretation for about 50% of the variant classified as “likely pathogenic.” These results reinforce the idea that a functional validation of VUS is essential before inferring a level of pathogenicity to specific genetic variants.

Finally, the latest advances in targeted genetic engineering, especially using CRISPR-CAS9, allow researchers to directly mimic patient-specific missense mutations onto the endogenous zebrafish gene. Indeed, homologous recombination events can occur by adding a nucleic acid donor template to the CRISPR cocktail to be microinjected. Although, due to technical limitations, the overall efficiency of this knock-in application remains much lower as compared to the generation of knockout mutations, the generation of such patient-specific genetic avatars has been accomplished in TDP43/ALS (Armstrong et al., 2016), in *FBN1* in various heritable connective tissue disorders (HCTD) (Yin et al., 2021), RPS14 in myelodysplastic syndrome (MDS) (Ear et al., 2016). One of the limitations of this approach is that the protein residue that is the subject of the mutation may not be conserved in zebrafish. Additionally, it is possible that although an orthologous gene is present in the zebrafish genome for a particular study, specific missense mutations may not lead to the same effects in a human or fish protein. An alternative method would necessitate recombining, at the endogenous locus, the whole human coding DNA as a transgene, but such genetic engineering would be time-consuming. Interestingly, a recent work using nematode worms describes such a “humanized” functional assay by developing an *in vivo* platform for screening the pathogenicity of VUS in the *STXBP1* gene associated with epileptic syndromes (Zhu et al., 2020). In this study, mutations were introduced by CRISPR-Cas9 and modeled using *Caenorhabditis elegans* to mimic and investigate the pathogenicity of gene variants. This illustrates, once again, how simple *in vivo* assays could quickly and accurately determine if a VUS is pathogenic or benign and how this could be applied to zebrafish.

In summary, *in vivo* zebrafish studies can provide an additional line of biological evidence to bridge the gap between variant identification and their pathogenic classification. Zebrafish can be considered an attractive multi-assay platform to characterize the pathogenicity of specific genetic mutations. However, the experimental approach must be defined according to the type of mutation apprehended (loss-of-function vs. gain-of-function; Figure 1). Specific technical limitations may hinder the standardization of these functional approaches *in vivo*.

## Using Zebrafish to Accelerate Drug Discovery

**FIGURE 2:**
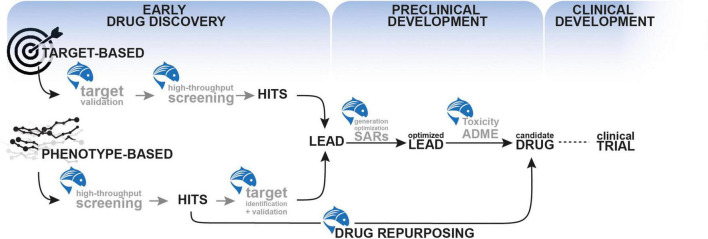
The use of zebrafish as a predictive preclinical model to accelerate various stages of drug development, from early discovery to preclinical development.

Drug discovery is a complex and lengthy process that entails years of meticulous planning, from the initial discovery of an active ingredient (drug-like compound) to the development process, which includes testing in animal models and finally in humans (Zon and Peterson, 2005). On average, it takes 10–15 years and US$2.6 billion for an active ingredient to reach the bedside (Seyhan, 2019) and this is primarily due to the failure of several candidate compounds at various stages of the drug discovery timeline (Bhusnure et al., 2015). Although *in vitro* and *in vivo* mammalian models are used to lower the cost and time of drug discovery, *in vitro* studies are less human-translatable and mammalian models make the entire process time-consuming, expensive, and laborious. Zebrafish can bridge the gap between *in vitro* studies and rodent models due to easy maintenance, cost-effectiveness, and reduction in the number of animals employed in regulatory testing according to the 3Rs (replace, reduce, refine) (Fleming and Alderton, 2013; Geisler et al., 2017). They also enable an early prediction of *in vivo* toxicity and safety data, which reduces the likelihood of drug failure later on. This section of the review will discuss how zebrafish can be used as a predictive preclinical model to accelerate various stages of drug development, from early discovery to preclinical development (Figure 2).

### Early Drug Discovery

The end goal of the early drug discovery phase is to identify an active compound that has the potential to develop as a drug. Zebrafish can be utilized in the early stages of drug development using two primary methodologies: target-based and phenotype-based approaches.

The starting point of target-based approaches is a defined molecular target that is hypothesized to have an essential role in disease. Such target-centric methods have been the dominant approach to drug discovery in the pharmaceutical industry. However, the process of target *validation* is complex and associated with a high degree of uncertainty and failure. Thus, target identification is critical in this process, and selecting a relevant target requires a greater understanding of the disease’s pathophysiology and molecular process (Gashaw et al., 2012). As zebrafish have been successful in mimicking a plethora of neurological disorders (Ramesh et al., 2010; Khan et al., 2017; Fontana et al., 2018; Gawel et al., 2020; Razali et al., 2021; Wang et al., 2021), it can pave the way for the discovery of such novel drug targets. One classical approach to target identification in zebrafish has been the use of morpholino-oligonucleotides but as said previously, their popularity and the confidence in their specificity has declined. Only a limited number of studies to date have used novel targeted-mutagenesis techniques such as CRISPR/Cas9 to generate target discovery studies in the neuroscience field. However, it is expected that a growing number of studies will be reported soon (Rubbini et al., 2020) as shown by the example of target identification in a *C3orf70* knockout zebrafish mutants with impaired circadian rhythm and altered light-dark neurobehaviors (Ashikawa et al., 2019) in which the *C3orf70* gene was reported to be a shared target of *Neurog1/2* and *Asc11*. As a result, *C3orf70* mutations may be linked to neurodevelopmental and neuropsychiatric diseases in these brain locations and could be exploited as a therapeutic target (Ashikawa et al., 2019). Target identification can also arise from exploratory transcriptomics investigation on a genetic zebrafish model for a particular disease. For instance, our group developed a zebrafish model of glycine encephalopathy carrying a mutation in a glycine metabolism enzyme (*GLDC*: glycine decarboxylase) (Riche et al., 2018). By whole transcriptome analysis of larval mutant brains, our study revealed changes in essential genes regulating the synaptic clearance of glycine, such as glycine transporter 1 (*glyt1*). Such a reduction of expression of this transporter exacerbates the accumulation of glycine at the synapse, thus the neurometabolic phenotype. Following target identification, the next step is to validate its involvement in the pathogenicity of the disease of interest to confirm its relevance and pave the way for defining essential properties that future hit compounds targeting it must fulfill. In the example of glycine encephalopathy, we demonstrated that boosting the expression of *glyt1* in mutant zebrafish embryo by mRNA microinjection was able to rescue the early motor phenotype associated with the mutation (Riche et al., 2018). Therefore, *glyt1* appears as an interesting novel target in the context of this disease. The use of zebrafish for target validation is also exploited by several contract research organizations (CROs) that propose to perform standardized functional assays on a target of interest (BioBide, ZeClinics, INVENesis, *InVivo* Biosystems). However, target-based screening for CNS disorders is more challenging due to the multifactorial nature of many neurological diseases.

Unlike target-based techniques, *in vivo* phenotype-based drug discovery allows the identification of compounds that modify the disease phenotype with no prior knowledge of a particular target. Notably, such target-agnostic approaches in the whole organism can later identify novel therapeutic targets. Currently, zebrafish is widely used as an *in vivo* phenotypic screening platform and has led to the development of several successful therapeutics. As a first step, phenotypic screening aims to identify a so-called “hit” compound with the desired effect on the phenotype screened. Thanks to its small size, zebrafish larvae can be placed in multi-well plates for screening the impact of various molecule libraries (including bioactive compounds, commercially available chemical libraries, and natural compounds) on a specific phenotype such as behavioral tracking, gene expression assay, or fluorescent reporter studies (Mills and Gallagher, 2017; Deakin et al., 2019; Abdelmoneim et al., 2020). Simple motor phenotypes can be assessed, such as a simple photo motor response assay using zebrafish embryos that allows screening for different profiles of psychotropic compounds in a high-throughput fashion (Kokel et al., 2010). In a *sod1*-mutant zebrafish model of Amyotrophic lateral sclerosis, McGown et al. (2016) developed a high throughput screening assay using a fluorescence-based readout of neuronal stress. Another commonly used behavioral assay is the increased motility response induced by exposure to Pentylenetetrazole (PTZ), a pro-convulsant drug (Baraban et al., 2005; Winter et al., 2008). Such an assay has been used to identify anti-seizure compounds *in vivo* in zebrafish. For instance, Baxendale et al. (2012), screened approximately 2,000 compounds and identified 46 hit molecules that suppressed PTZ-induced seizure phenotype in zebrafish larvae. Moreover, Kim and colleagues performed a screen of 1,403 bioactive compounds using an *in vivo* whole-organism screening assay by imaging dopaminergic neurons of larval zebrafish in a high throughput manner. In short, the authors used a transgenic zebrafish model in which they induced dopaminergic neuron loss. Using an automated imaging microscope, they screened the effect of each compound on the survival of this neuronal population *in vivo*. Their study identified several hit molecules that significantly protected dopaminergic neurons in this assay (Kim et al., 2022). These examples show how zebrafish can identify hit compounds unbiasedly using phenotypic screening. Once a list of hit molecules is in hands, the next step is to further evaluate them based on more selective screening criteria to select the “lead” molecules that are more likely to be therapeutically valuable (hit-to-lead phase, Figure 2). Although zebrafish have been extensively used for hit identification using high-throughput screening, it is less frequently used to further characterize and optimize lead candidate compounds. However, zebrafish genetic models could be used to correlate phenotypic readouts with target binding and could therefore be a convenient platform for the optimization of lead candidates (lead expansion phase). One can imagine that the classical way of thinking surrounding more advanced therapeutic development necessitates the use of mammalian models. However, in the next section, we will see that zebrafish can autonomously bridge the gap between bench and bedside.

### Drug Repurposing

As we discussed previously, zebrafish are convenient for phenotypic screening, and the effect of thousands of compounds can be tested on various disease-relevant phenotypes. Such molecule libraries can contain already approved drugs for which a novel therapeutic indication could be unveiled. Repurposable drugs have a low risk of failure since their toxicology profiles have been thoroughly determined in previous clinical studies and are known to be safe in humans (Ayyar and Subramanian, 2022). Using zebrafish, phenotypic screening of over 3,000 commercially available FDA-approved drugs has been conducted on a genetic zebrafish model of Dravet syndrome, a severe infantile epilepsy (Baraban et al., 2013; Dinday and Baraban, 2015; Sourbron et al., 2016). In this work, the authors identified several 5-HT serotonin-receptor agonists as being able to suppress spontaneous seizures in mutant larvae. The phenotypic assay was conducted on a behavioral seizure assay and then confirmed using brain electrographic recordings (Baraban, 2013). Based on these results, they jumped straight from tank to bedside by treating pharmacoresistant Dravet syndrome patients with lorcaserin, a clinically approved serotonin receptor agonist (compassionate use) (Griffin et al., 2017). Interestingly, they observed reductions in seizure frequency and severity, thus proving the relevance of targeting the serotonin receptor pathway in Dravet syndrome (Griffin et al., 2017). Among the hit compounds they identified, clemizole, a classical antihistaminic agent, was found particularly efficient in preventing seizures. Further protein binding profiling showed that clemizole could act as a serotonin receptor antagonist, and a spin-off biotech (EpyGenix Therapeutics) is currently developing clemizole (EPX-100) and derivatives (EPX-101, EPX-102, and EPX-103) for the treatment of Dravet syndrome.

Another example of a successful drug-repurposing screen is the work of Patten and colleagues, who performed a chemical screening of 3,850 small molecules on *C. elegans* models of Amyotrophic Lateral Sclerosis (ALS). This initial screen identified 13 hits compounds further tested in transgenic zebrafish expressing mutant TDP-43. The authors assessed the effects of these hit molecules on multiple phenotypes from swimming motility, known to be reduced in mutant-TDP43 expressing transgenic fish, orphan neuromuscular synapses and electrophysiology recordings of synaptic transmission (Patten et al., 2017). They showed that one hit molecule, pimozide, was particularly effective at alleviating these phenotypes. They further confirmed the neuroprotective effect of pimozide on two other zebrafish ALS models expressing FUS(R521H) and SOD1(G93A) based on motor phenotypic readouts (swimming response, swim duration, distance swam, and maximum swim velocity). Finally, after confirming the positive effect of pimozide on stabilizing the neuromuscular neurotransmission in a genetic mouse model of ALS, the authors initiated a short randomized controlled trial of sporadic ALS patients. Remarkably, this small-scale phase 2A trial demonstrated stabilization of motility and evidence of target engagement at the neuromuscular junction in ALS patients (Patten et al., 2017). A phase II randomized, placebo-controlled, double Blinded, multi-centered phase 2B clinical trial is ongoing to confirm the positive effect of pimozide in 100 ALS patients (NCT03272503). Interestingly, as a neuroleptic, pimozide specifically targets dopamine D2 receptors. Still, the authors showed that its effect on neuromuscular junctions relies on the antagonism of T-type Ca2 + channels, one off-target property of pimozide. Thus, the main accomplishment of this work does not solely rely on the discovery of a new therapeutic compound for ALS (the first such accomplishment in nearly two decades) but also on the unveiling of a novel pathogenic mechanism that can be further harnessed for the development of new therapeutics targeting T-type calcium channels.

These studies illustrate how zebrafish can be effectively used in early drug discovery processes from hit identification, hit-to-lead selection and target discovery and validation (Figure 2).

### Preclinical Studies

Once a lead compound is identified from the early drug discovery phase, it usually undergoes optimization through structure-activity relationship (SAR) profiling. These preclinical studies aimed to refine the lead molecule’s structure and apprehend the lead molecule’s safety *in vivo* while improving its adsorption properties (Temml and Kutil, 2021). There is an interest in incorporating these lead optimization essays as early as possible during the drug discovery process to increase the chance of success while reducing the costs associated with failure later on. Zebrafish is highly amenable to studying such structure-activity relationships (SARs) and therefore participate in predicting the biological activity of compounds based on their molecular structure and improve the development of structural analogs with better activity. Indeed, several SAR studies have been performed in zebrafish, where the effect of different structural analogues (first obtained from *in vitro* studies, such as molecular docking or ligand-receptor binding studies) is tested on zebrafish behavioral phenotypes as readouts. Coming to the example of clemizole that has been identified using zebrafish chemical screening for Dravet syndrome (discussed above), SAR studies have been conducted on 28 newly synthesized analogues of clemizole with varying 5-HT2R binding affinities (Griffin et al., 2019). The authors identified three analogs, specifically binding to 5-HT2B receptors, exerting a potent suppression of convulsive swim behavior and electrographic seizure activity in *scn1lab* zebrafish models of Dravet syndrome (Griffin et al., 2019).

Another example is the use of zebrafish to perform SAR studies for an antipsychotic compound in the context of schizophrenia (Hellman et al., 2020). In this study, Hellman et al. aimed at developing analogs of N-Desmethylclozapine (NDMC), the primary metabolite of clozapine that is the leader of the so-called “atypical” or second-generation antipsychotics. NDMC can act as muscarinic M1 receptor agonists, and this activity is associated with improvement in cognitive functioning in patients. However, NDMC failed phase 2 clinical trials on schizophrenic patients. Thus, the development of NDMC analogs with enhanced M1 receptor agonist functions might be promising. Using *in vivo* behavioral response profiles in zebrafish, they evaluated the antipsychotic efficacy of several NDMC analogs they developed and identified one of them that demonstrated antipsychotic activity similar to clozapine, including M1 agonist activity. Thus, thanks to this SAR study using *in vivo* phenotypic zebrafish readout, they identified one interesting NDMC analogue suitable for further development as an antipsychotic compound with potential procognitive activity (Hellman et al., 2020).

The next step after optimizing leads is to assess the toxicity profile of the lead molecule. Indeed, many lead compounds fail later in the development phase due to toxicity and efficacy concerns. Mainly, neurotoxicity is one of the significant attritions in drug development. Here again, zebrafish can be used as a predictive preclinical model to rapidly eliminate hazardous compounds and prioritize compounds for further clinical studies (McGrath and Li, 2008). The most common neurotoxic endpoints are alterations in zebrafish neurobehavioral when exposed to various toxins or chemicals. Several neurotoxicity assays are performed using zebrafish behavior phenotypes as readouts obtained from locomotor tests, photo motor tests, touch response, and acoustic tests (Polaka et al., 2022). The photo motor response assay, which involves the automatic tracking of larval movement in response to various lighting conditions, is widely used for neurotoxic screening (Cassar et al., 2020). For example, Knecht et al., used a larval photo motor experiment to test the neurotoxic effects of benzo[a]pyren. This ubiquitous environmental pollutant may contribute to human cancer development (Knecht et al., 2017). Other studies showed that the zebrafish embryotoxicity test [ZET, OECD236 (Busquet et al., 2014; Braunbeck et al., 2015)] could accurately predict the toxicity of known developmental neurotoxicant substances (Beker van Woudenberg et al., 2013).

Moreover, the zebrafish-based locomotor activity effectively classified well-known compounds as neurotoxic or non-neurotoxic, which were 90% identical to prior findings from mammals (Selderslaghs et al., 2013). Zebrafish locomotor activity can also be evaluated by touch-evoked response tests, which record zebrafish larvae’s behavior in response to a tactile stimulus applied to the head or tail as a measure of sensory and motor integration (d’Amora and Giordani, 2018). The neurotoxic effects of insecticides such as endosulfan I and endosulfan sulfate were confirmed in zebrafish (Stanley et al., 2009). Finally, as said before, one main advantage of performing preclinical studies in zebrafish is that it benefits from combining SAR, toxicity and ADME (absorption, distribution, metabolism, and excretion) studies. ADME studies aim at studying the fate of an active substance contained in a drug after its administration in the body, including its absorption (A), distribution (D), metabolism (M), and excretion, including its metabolites (E). However, a key hurdle for this model is the ability to determine the effective compound concentration in the zebrafish and to correlate this dose with rodent and human data. Indeed, drug exposure remains constant as the larvae are immersed in bathing media containing the drug; thus, quantifying drug uptake into zebrafish larvae remains the main limitation. However, several techniques are being developed to accurately quantify compound uptake in larvae using mass spectroscopy or NMR to analyze the drug absorption and distribution, which are analyzed on whole embryos or specific organs or tissues (Zeclinic). Moreover, researchers from KU Leuven are developing analytical methods to measure the whole-body uptake of compounds in 10-day-old zebrafish larvae using ultra-high-performance liquid chromatography (UHPLC) (Kislyuk et al., 2017). Their data showed that a single zebrafish could be used to study the whole-body uptake of a particular drug. Then a similar methodology can be used to learn the uptake of pharmaceuticals in the brain of zebrafish and hence explore the potential of zebrafish as a predictive blood-brain-barrier model (Kislyuk et al., 2018). Moreover, using these techniques, the concentration of compounds in the incubation water can be compared to the concentration in embryos to get valuable insights into drug metabolism and excretion. In this case, metabolites, when known, may also be analyzed both in embryos and incubation water.

As a result, the shared pharmacology between zebrafish and humans makes zebrafish an important preclinical model that helps accelerate the drug discovery process at multiple levels (Figure 2). However, one significant limitation of the use of zebrafish as a model in early drug discovery relates to the development of the blood-brain barrier (BBB). BBB is a complex structure that represents a physical blockade for drugs to access the CNS. Thus, BBB permeability needs to be carefully taken into consideration while testing the efficiency of therapeutic compounds for brain disorders. In zebrafish, the BBB starts to form at 3 days post-fertilization but its maturation progresses until 10 dpf (Fleming et al., 2013). It is important to note that during this maturation period, during which most screening experiments are usually performed (e.g., 5 dpf), the zebrafish BBB has been described as “leaky.” Thus, caution should be exercised with interpretations of BBB crossing when testing compounds on zebrafish larvae at stages when the BBB is still permeable.

## Using Zebrafish to Predict Treatment-Responsiveness and Tailor Medication

### Choosing the Best Available Treatment

As discussed in the previous part of this review, zebrafish is a convenient tool for identifying new lead molecules for therapeutic purposes in neurosciences, especially epilepsy. Indeed, the similarity of brain seizures between zebrafish and humans makes this model particularly relevant for translational research perspectives. However, there is also a need to identify which drug is more likely to be efficient for a particular group of patients among the list of available treatments. Indeed, although there are 28 classical Antiepileptic Drugs (AEDs) available to epileptic patients, treatment-response is often unpredictable, and approximately one-third of patients fail to gain complete seizure control with pharmacotherapy alone (Chen et al., 2018). Moreover, determining the best AED among the 28 classical ones mostly relies on an empirical trial-and-error method from medical doctors. More importantly, it has been shown that an inappropriate first medication can have severe consequences on the efficacy of further treatments (Pawluski et al., 2018). This underlines the need to identify which AED works best for each patient as quickly as possible. In the last decades, the genetic component of many epilepsies has been unraveled, and it helped better classify the different types of epilepsies depending on their genetic etiology. In line with these discoveries, many zebrafish genetic mutants have been generated carrying mutations in different genes associated with brain seizures and epileptic syndromes (Baraban, 2007; Hortopan et al., 2010; Stewart et al., 2014; Griffin et al., 2018; Gawel et al., 2020). Our group participated in this effort by developing several zebrafish lines carrying loss-of-function mutations in epilepsy-causing genes such as *gabra1* (Samarut et al., 2018), *gabrg2* (Liao et al., 2019), *scn1lab* (unpublished), and *depdc5* (Swaminathan et al., 2018). These mutant fish undergo brain seizures either spontaneously or under stress conditions, and the treatment-responsiveness of these lines, measured unbiasedly through behavioral and/or brain activity recording readouts, recapitulates drug response in patients. This is consistent with a recent review showing that zebrafish models of Dravet syndrome are particularly reliable in pharmacological and clinical relevance (Griffin et al., 2018). The anti-seizure effect of well-known AEDs, such as valproic acid, carbamazepine, gabapentin, diazepam, lacosamide, and pregabalin, has been tested on PTZ-induced seizures in zebrafish (Baraban et al., 2005; Berghmans et al., 2007; Gupta et al., 2014). Interestingly, in these assays, many non-GABAergic drugs also prevented PTZ-induced seizures, which broadened the scope of PTZ screening in zebrafish compared to rodent models. During this study, the efficiency of AEDs has been correlated with different types of seizure-like behaviors. For instance, valproic acid, gabapentin, lacosamide and carbamazepine showed a concentration-dependent increase in latency at all stages of seizures, which was significant for valproic acid at 300 μM to 10 mM, gabapentin at 1-10 mM, lacosamide at 100 μM to 3 mM and carbamazepine at 10–100 μM, while pregabalin failed to increase in seizure latency at all the stages compared with the control group. Other drugs showed saturated responses, such as gabapentin at > 1 mM or diazepam at 10 μM. These examples show how testing the effectiveness of different drugs on relevant fish models can help develop personalized medicine approaches, particularly in epilepsy. Indeed, by applying these standardized screening techniques to more chemical and/or genetic models of epilepsy, we could better correlate genotypes to treatment-responsiveness and translate these findings to patients by better predicting which drug is the more likely to be efficient depending on the genetic etiology of diseases. However, even at the level of a single disease such as epilepsy, the spectrum of phenotypes and the genotype-phenotype correlations are very complex since different mutations, even at the level of the same gene, can lead to different types of seizures (Johannesen et al., 2016; Kang and Macdonald, 2016; Gontika et al., 2017; Shen et al., 2017). As a result, each epileptic patient might have to be considered unique in treatment responsiveness. Thanks to the latest improvements in targeted genome editing techniques, we could foresee the future development of patient-personalized zebrafish genetic avatars that could tailor the treatment at the level of individual patients/mutations. More broadly, this emphasizes the need for better studying how variations of DNA and RNA characteristics are linked to an individual’s response to medication. This is the exact definition of pharmacogenomics (PGx), another important example of the field of precision medicine, which combines pharmacology and genomics to develop effective, safe medications that can be prescribed based on a patient’s genetic fingerprint. Thus, PGx has the potential to revolutionize the practice of medicine by individualizing treatment through the use of novel diagnostic approaches to predict predicting which patient will particularly benefit from a medication, which one will not respond at all, and which will experience significant negative side effects (Topic, 2008). In most cases, Single Nucleotide Polymorphisms (SNPs) are the key to a better understanding an individual’s response to treatment and potential risks (Alwi, 2005; Katara, 2014). These single nucleotide changes may occur in non-coding and coding regions of the genome. This creates a broad range of genetic diversity among the population. They are also called “genetic fingerprints,” which pave the way for establishing new diagnostic tools and further PGx development for individuals. Promising gene-based methods aiming at improving precision in psychotropic medication allowed the identification of specific genetic polymorphisms in genes involved in the pharmacokinetics and pharmacodynamics of psychiatric drugs (Malhotra et al., 2004; Malhotra et al., 2007; Steimer, 2010; Lisoway et al., 2021). This can bring valuable information for tailoring treatment for anxiety, bipolar disorder, schizophrenia, or ADHD based on the patient’s specific genetic profile (Hamilton, 2015; Kose and Cetin, 2018). However, neurology may be lagging in the PGx field behind other specialties such as oncology or immunology include the heterogeneity of disorders and the lack of biomarkers. Moreover, the complex variety of pathogenic mechanisms makes psychiatric disorders particularly challenging to treat with a vague definition and standardization of clinical outcomes among cohorts of patients. That creates an excellent opportunity for researchers to fill this gap with the use of relevant biological models. Despite the advantageous genetic and pharmacological accessibility of zebrafish, it has not been exploited widely in pharmacogenomics. However, its potential in identifying genetic determinants of the physiological response to anesthetic drugs has been recently reviewed by Bedell et al. (2018). Several complex behavioral assays are available to study drug response in zebrafish, including the photo motor startle response (PMR). PMR does not involve any visual organs and is one of the earliest forms of motor behavior in zebrafish (between 30 and 40 h post-fertilization) (Kokel et al., 2010; Kokel et al., 2013). Interestingly, it has been shown that this early behavior is altered in the presence of neuroactive compounds and anesthetics (Copmans et al., 2016; Gauthier and Vijayan, 2018). Therefore, it can be used for high-throughput chemical and/or genetic screens to identify modulators of a variety of drugs, including anesthetics and other neuroactive compounds. Considering the latest progress made in mimicking precise genetic conditions in zebrafish, it is a model well-positioned to investigate the genetic aspects of drug response *in vivo*. This is what Yang et al. (2019) have been proving by testing the sensitivity of zebrafish mutants lacking the expression of specific γ-aminobutyric acid type A (GABAA) receptors subunits to anesthetics. However, the teleost-specific whole-genome duplication that occurred during evolution led to more genes within the zebrafish’s genome that can complexify such studies (Sato and Nishida, 2010). Indeed despite the strong genetic similarity between zebrafish and humans, the functional redundancy between paralogues genes can make mimicking patient-specific SNPs challenging and dampen the translatability of pharmacogenetics studies performed in zebrafish to humans.

## Conclusion

**FIGURE 3:**
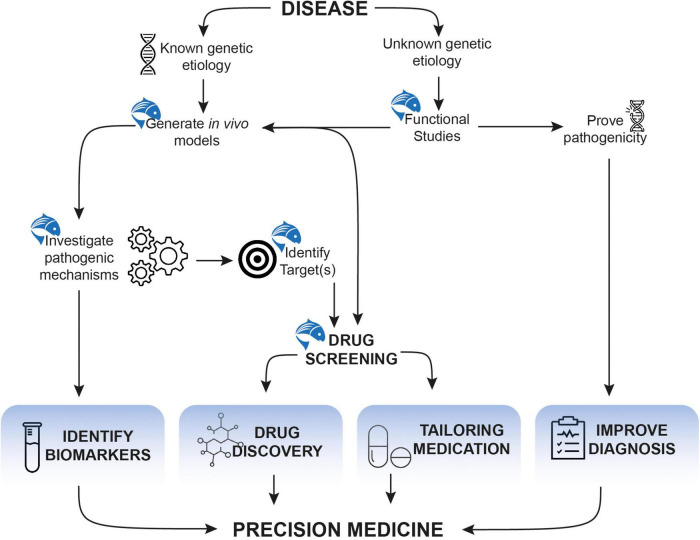
Accelerating the development of precision medicine approaches using zebrafish.

The popularity of zebrafish as a model is well-established as it is a formidable tool in the field of developmental biology, genetics, and pharmacology. Its use fits at multiple impactful levels of the broad precision medicine framework (Figure 3). Its success, popularity and utility will continue to grow as novel genetic engineering and innovative screening techniques will continue to emerge.

## Author Contributions

ÉS designed the figures. All authors wrote and reviewed the manuscript and approved the submitted version.

## Conflict of Interest

ÉS was a co-founder of Modelis Inc. The commercial affiliation did not play any role in this study; in particular, it did not have any additional role in the study design, data collection and analysis, decision to publish, or preparation of the manuscript. The remaining authors declare that the research was conducted in the absence of any commercial or financial relationships that could be construed as a potential conflict of interest.

## Publisher’s Note

All claims expressed in this article are solely those of the authors and do not necessarily represent those of their affiliated organizations, or those of the publisher, the editors and the reviewers. Any product that may be evaluated in this article, or claim that may be made by its manufacturer, is not guaranteed or endorsed by the publisher.
